# A Novel Variant in *SYNE4* Confirms its Causative Role in Sensorineural Hearing Loss

**DOI:** 10.4274/balkanmedj.2017.0946

**Published:** 2018-03-15

**Authors:** John Masterson, Busegül Yıldırım, Ece Gökkaya, Suna Tokgöz Yılmaz, Mustafa Tekin

**Affiliations:** 1 John P. Hussman Institute for Human Genomics, University of Miami Miller School of Medicine, Miami, USA; 2 Department of Audiology, Ankara University School of Medicine, Ankara, Turkey; 3 Dr. John T. Macdonald Foundation, University of Miami Miller School of Medicine, Miami, USA

**Keywords:** Hearing loss, *SYNE4* gene, high-throughput DNA sequencing

## Abstract

**Background:**

Hearing loss is the most common sensory deficit with many genetic and environmental underpinnings. While causative DNA variants have been identified in over 100 genes, most deafness-causing variants are rare, apart from a few exceptions. A single *SYNE4* variant co-segregating with hearing loss has recently been reported in two Middle-Eastern families.

**Case Report:**

In this report we present two members of a family with non-syndromic high frequency sensorineural hearing loss who are homozygous for a novel pathogenic *SYNE4* variant c.129-1G>T.

**Conclusion:**

This case report provides supportive evidence for the causative role of *SYNE4* variants in hearing loss by presenting an additional family with a novel DNA variant.

Hearing loss (HL) is the most frequently occurring of all congenital sensory deficits; displaying an incidence of 0.1-0.2% in the newborn population (1). While HL has many known genetic and environmental causes, genetic aetiologies predominate in the developed world (2). Genetic or hereditary HL can present as part of a larger syndrome or have a non-syndromic phenotype where HL is the only clinical symptom. Previous studies have elucidated that the genetic transmission of non-syndromic HL is autosomal recessive in 75-85% of all cases, and autosomal dominant in 15-25% of cases, with a small proportion of cases showing X-linked or mitochondrial inheritance (1-2%) (3).

More than 140 loci have been found to be associated with non-syndromic HL. There have also been over 400 HL syndromes described in the Online Mendelian Inheritance in Man database which show a distinct constellation of clinical features including HL (4,5).

Despite this large number of identified mutations, roughly half of all cases of non-syndromic congenital deafness in certain populations can be traced to a single gene: *GJB2*. The remaining deafness-causing mutations are rare and many are typically found in only a single, or very few families (6). The goal of this report is to identify the genetic aetiology of deafness in individuals with rare forms of non-syndromic sensorineural HL. A novel variant in a rare deafness-causing gene (*SYNE4*; MIM: 615535) was identified and helps to clarify the genotypic profile of this uncommon form of sensorineural HL.

## CASE PRESENTATION

The study was approved by the IRB at the University of Miami and the Ethics Committee of Ankara University. Blood samples were collected after informed consent was obtained. We evaluated a family (family 951) consisting of a brother and sister (from the same mother and father) with HL born to Turkish parents who are first cousins.

**Figure 1:**
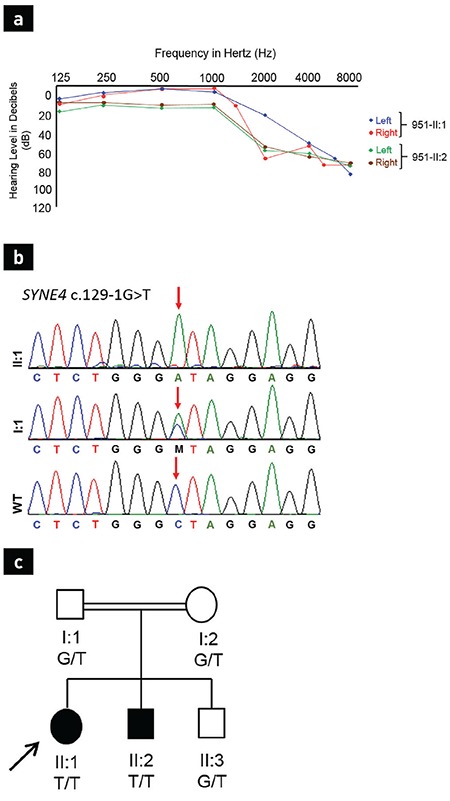
Representation of the Genotype-Phenotype information of the family. Audiogram for individual I:1 and I:2 (a). Electropherogram of the *SYNE4* c.129-1G>T for individual I:1 and II:1 (b). The pedigree and segregation of the variant in the family (c).

Individual II:1, the proband, is a 13-year-old female born after an uncomplicated pre- and neonatal period. She has bilateral post-lingual HL that was diagnosed three months prior to examination. She had no earache, ear discharge, or ear congestion, and no sense of pressure in the ear or dizziness. An audiogram performed at the time showed moderate bilateral sensorineural HL with a significant loss of higher frequencies (>1 kHz; Figure 1a). Inner ear and temporal magnetic resonance imaging (MRI) studies were normal. An electrocardiogram (EKG) and echocardiogram were unremarkable. A thorough physical exam including an ophthalmoscopy revealed no abnormalities. 

Individual II:2, the 11-year-old brother of II:1, was also noted to have HL two months prior to examination. He complained of hearing impairment in high-pitched sounds. He denied any earache, discharge, or congestion, and there was no sense of pressure in the ear or dizziness. An audiogram performed at the time showed a moderate bilateral sensorineural HL with sudden decreases in 1 kHz and 2 kHz. Inner ear and temporal MRI, echocardiogram and EKG were unremarkable.

### Whole exome sequencing

Whole exome sequencing (WES), variant filtering/interpretation and Copy Number Variant analysis were performed in the proband to identify potential causative variants using previously published protocols (6). Sanger sequencing was performed for the confirmation and co-segregation of candidate variants.

WES revealed that the proband of family 951 was homozygous for the *SYNE4* NM-001039876.1:c.129-1G>T variant (Table 1). This variant was confirmed by Sanger sequencing (Figure 1b). The affected brother was also shown to be homozygous and both parents were heterozygous for the variant (Figure 1c).

## DISCUSSION

HL is the most common sensory disorder which displays extreme genetic heterogeneity. In this case study, we identified a novel variant in *SYNE4* causing HL.

*SYNE4* encodes nesprin-4 (Nesp4), which localises to the outer nuclear membrane and is part of the linker of nucleoskeleton and cytoskeleton (LINC) complex in the nuclear envelope. On one end, Nesp4 attaches to the protein Sun1, another member of the LINC complex, which is anchored beneath the inner nuclear membrane and spans the perinuclear space. On the opposite end, Nesp4 attaches kinesin in the cytoplasm, effectively forming a bridge between the nuclear membrane and the cytoskeleton of the cell, allowing the nucleus to be securely anchored within the cytosol. This complex plays an essential role in allowing cell nuclei to localise to a specific region within the cytosol, and to relocate within the cell as necessary, which is essential for cell function (7).

Human outer hair cells, which have a columnar morphology, show preferential nuclear localisation to the basal part of the cell. These cells are extremely sensitive to environmental stressors and lack the ability to regenerate and their degeneration results in permanent HL that cannot be recovered. Proper function of outer hair cells is intrinsically tied to their motile nature and ability to change length upon membrane hyperpolarisation and depolarisation (8). It is therefore reasonable to predict that a compromised connection between the nucleus and cytoskeleton would be detrimental to the proper transmission of mechanical signals from the cell to the nucleus and therefore impact proper cell function.

In a 2013 publication, Horn et al. (9) identified progressive high-frequency HL (MIM: 615540) in two consanguineous families of Iraqi Jewish ancestry with a homozygous deletion mutation, c.228delAT, in *SYNE4*, presumably rendering the product protein non-functioning. As part of the same publication, knockout mice of *SYNE4* showed apical -as opposed to the normal basal- localisation of the nuclei and degeneration of outer hair cells as hearing matured, leading to progressive HL. To affirm that a wholly intact LINC complex is essential to outer heir cell survival, Horn et al. (9) also created a SUN1 knockout mouse model, removing the protein to which Nesp4 connects. These mice demonstrated an identical phenotype to the *SYNE4* knockout mice (9).

Affected members of family 951 mirrored this clinical presentation of progressive, high-frequency HL, lending further support to the association of deafness with the *SYNE4* variant. Additionally, the novel variant identified in family 951 is a splice-site mutation. According to the 2015 The American College of Medical Genetics and Genomics (ACMG) Standards and Guidelines, this mutation has very strong evidence of pathogenicity as it is located at the canonical-1 splice site, where a loss of function is a known mechanism of disease (10), as reported by Horn et al. (9). The ACMG also recommends the use of multiple computational predictive programs to assess the pathogenicity of missense variants. The variant in family 951 was predicted to be disease causing by both Mutation Taster and CADD with a score of 24.3 (Table 1). Furthermore, when compared to the deletion mutation identified by Horn et al. (9), the mutation in family 951 provides genotypic heterogeneity within the similar clinical presentation shown by *SYNE4* dysfunction in these families.

Identification of this novel variant in *SYNE4* further elucidates the genotypic profile of the rare deafness phenotype with which it is associated. Its identification also lends further support to the pathogenic role of *SYNE4* and fortifies the already growing list of causative variants of hereditary HL. Continual expansion of this database will allow physicians to more speedily and accurately identify culprit gene mutation variants in patients presenting with less common HL phenotypes.

## Figures and Tables

**Table 1:**
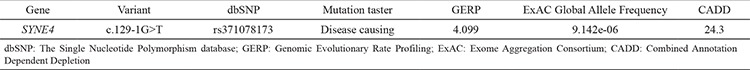
Characteristics of the *SYNE4* variant
